# A simple multiplex polymerase chain reaction assay for rapid identification of the common pathogenic dermatophytes: Trichophyton interdigitale, Trichophyton rubrum, and Epidermophyton floccosum

**DOI:** 10.18502/cmm.7.2.7030

**Published:** 2021-06

**Authors:** Sama Faramarzi, Marjan Motamedi, Ali Rezaei-Matehkolaei, Shima Aboutalebian, Saham Ansari, Mojtaba Didehdar, Mehran Bahadoran, Hossein Mirhendi

**Affiliations:** 1 Department of Medical Parasitology and Mycology, School of Medicine, Isfahan University of Medical Sciences, Isfahan, Iran; 2 Department of Medical Parasitology and Mycology, School of Medicine, Shiraz University of Medical Sciences, Shiraz, Iran; 3 Department of Medical Mycology, School of Medicine, Ahvaz Jundishapur University of Medical Sciences, Ahvaz, Iran; 4 Department of Medical Parasitology and Mycology, School of Medicine, Shahid Beheshti University of Medical Sciences, Tehran, Iran; 5 Department of Medical Parasitology and Mycology, School of Medicine, Arak University of Medical Sciences, Arak, Iran

**Keywords:** *E. floccosum*, *T. interdigitale*/*T. mentagrophytes*, *T. rubrum*, Dermatophyte, Multiplex PCR

## Abstract

**Background and Purpose:**

The most common etiological agents of human dermatophytosis in various parts of the world are *Trichophyton rubrum*, *Trichophyton interdigitale*, and *Epidermophyton floccosum*. The main aim of this study was to design and evaluate a simple and straightforward multiplex polymerase chain reaction (PCR) assay for reliable identification/differentiation of these species in clinical isolates.

**Materials and Methods:**

The reliable sequences of several molecular targets of dermatophytes species were used to design a multiplex PCR for the identification of common pathogenic dermatophytes. The isolates and clinical specimens examined in this study included seven standard strains of dermatophytes, 101 isolates of dermatophytes and non-dermatophyte molds/yeasts which had already been identified by sequencing or PCR-restriction fragment length polymorphism (RFLP), and 155 clinical samples from patients suspected of cutaneous mycoses.

**Results:**

Species-specific primer pairs for *T. rubrum* and *T. interdigitale*/*T. mentagrophytes* were designed based on the sequence data of the translation elongation factor 1-alpha gene, and the primers for *E. floccosum* targeted the specific sequence of the internal transcribed spacer region (ITS). The multiplex PCR successfully detected *T. rubrum*, *T. interdigitale*/*T. mentagrophytes*, and *E. floccosum* strains that were identified by sequencing or PCR-RFLP. However, the primer pairs selected for *T. interdigitale*/*T. mentagrophytes* cross-reacted with *Trichophyton tonsurans*. In testing the PCR system directly for clinical samples, the proportion of positive multiplex PCR was higher than positive culture (68.1% vs. 55.4%, respectively).

**Conclusion:**

The multiplex assay could detect three common agents out of several causal agents of dermatophytosis, namely *T. rubrum*, *T. interdigitale*, and *E. floccosum*. Therefore, by adding pan-dermatophyte primers it can be used as a comprehensive detection/identification test.

## Introduction

Dermatophytes are keratinophilic fungi and the most adaptable parasites of humans. They are the causes of dermatophytosis as the most superficial fungal infections with an estimated lifetime risk of 20–25% [ 1 ]. The predominant pathogenic species of dermatophytes vary within a geographical region and during different periods due to factors, such as population movement, socioeconomic circumstances, and the level of disease surveillance [ 2 ]. The most common etiological agents of dermatophytosis in the USA, Europe, and different parts of Iran are *Trichophyton rubrum*, *Trichophyton interdigitale*, and *Epidermophyton floccosum*, although other anthropophilic, zoophilic, or geophilic species of dermatophytes can cause infection [ 3 - 5 ]. 

Discrimination of dermatophytosis etiologic agents is important for the investigation of the epidemiological survey, as well as for therapeutic purposes [ 6 , 7 ]. In most mycology laboratories, these keratinophilic fungi are identified on the basis of gross examination of colonies from culture, microscopic examination of macro- and micro-conidia, growth requirements, and biochemical and physiological characteristics. However, these criteria alone may be insufficient since colonial features may be similar to other fungi or vary within a taxon. 

One of the prominent problems observed in mycology laboratories is distinguishing *T. rubrum* strains from members of the *T. mentagrophytes* species complex by using conventional diagnostic methods. These methods include hydrolysis of urea, *in vitro* hair perforation, pigment production, Tween opacity, sorbitol assimilation, and salt tolerance. Such traditional identification methods are labor-intensive, have poor sensitivity, require up to one week for fungal growth, and need significant expertise. Furthermore, sometimes, the same strains may show morpholo-gically diverse colonies, making the identification of the organism more difficult. This is especially true for *T. rubrum* and the members of the *T. mentagrophytes* complex when recovered from chronic infections by treatment with various antifungal agents as they often do not manifest their typical colonial morphology, pigmentation, and production of micro- and macro- conidia.

To overcome the defects of the classical methods and establish a simpler, more sensitive, and rapid system for routine use, several improvements, including molecular biological techniques, have been attempted for the identification of pathogenic fungi isolated from clinical specimens. Multiplex polymerase chain reaction (PCR) is widely used in the field of clinical microbiology as it allows simultaneous detection of more than one microbe [ 8 ]. This approach has been used to identify a variety of fungi, including dermatophytes [ 9 - 12 ]. However, despite their high frequency in human dermatophyte infections, a specific profile has not been reported for differentiating the three common species of *T. rubrum*, *T. interdigitale*, and *E. floccosum*. Therefore, the main aim of this study was to design and evaluate a simple and straightforward multiplex PCR assay for the reliable detection/differentiation of *T. rubrum*, *T. interdigitale*, and *E. floccosum* in clinical isolates.

## Materials and Methods

### Fungal strains and isolates

To optimize the specificity of the primers and the multiplex PCR, the present study was performed on the following: seven standard strains of dermatophytes, i.e., *T. rubrum* (CBS 288.86), *T. mentagrophytes* (CBS 318.56), *T. interdigitale* (CBS 130816), *Trichophyton erinacei* (CBS 344.79), *Trichophyton tonsurans* (CBS 120.65), *Trichophyton schoenleinii* (CBS 434.63), and *Arthroderma racemosum* (CBS 423.74) as well as 101 isolates of dermatophytes and non-dermatophyte molds/yeasts (Table 1) consisting of *Aspergillus niger*, *Mucor*, *Alternaria* sp., *Cladosporium* sp., *Trichosporon* sp., *Candida albicans*, and *Candida lusitaniae*. All tested dermatophytes were subjected to preliminary molecular identification by sequencing or PCR-restriction fragment length polymorphism (RFLP) as described previously [ 13 , 14 ].

**Table 1:** Summary of 101 multiplex polymerase chain reaction (PCR) results obtained from tested clinical isolates with PCR-restriction fragment length polymorphism (RFLP) or sequencing results

Clinical isolates	Species identified by multiplex PCR (n)	
Species identified by PCR-RFLP or sequencing (n)	*Trichophyton mentagrophytes*/* Trichophyton interdigitale* (24)	*T. mentagrophytes*/*T. interdigitale* (24)
	*Epidermophyton floccosum* (21)	*E. floccosum* (20)
		Negative (1)
	*Microsporum canis* (21)	Negative (20)
		*T. rubrum* (1)
	*Trichophyton rubrum* (16)	*T. rubrum* (15)
		*T. interdigitale*/*T. mentagrophytes*/*T. rubrum* (1)
	*Trichophyton tonsurans* (12)	*T. mentagrophytes*/*T. interdigitale* (10)
		Negative(2)
	*Aspergillus niger* (1)	Negative (1)
	*Mucor* (1)	Negative (1)
	*Alternaria* (1)	Negative (1)
	*Cladosporium* (1)	Negative (1)
	*Trichosporon* (1)	Negative (1)
	*Candida albicans* (1)	Negative (1)
	*Candida lusitaniae* (1)	Negative (1)
Total	101	101

### Clinical samples

In total, 155 samples (skin scrapings (n=83), nails (n=60), and hair (n=12)) were collected from patients suspected of cutaneous fungal infection. It should be noted that 110 samples were divided into three portions: a portion was examined microscopically in 10% KOH for the presence of fungal elements, another portion was cultured on Sabouraud dextrose agar (Biolife, Italy) supplemented with 40 mg l-1 chloramphenicol and 500mg l -1cyclohexemide and incubated at 27 °C for up to 4 weeks, and the third portion was used for DNA extraction and PCR analysis. 

### DNA isolation

The DNA was extracted from the fungal colonies and purified as described previously [ 15 ]. Briefly, 10–20 mm^3^ of the fresh colonies were added to the 1.5 ml tubes containing 300 μl of glass beads (0.5 mm in diameter), 300 μl of lysis buffer (100 mM Tris, pH 8; 10 mM EDTA; 100 mM NaCl; 1% sodium dodecyl sulfate [SDS]; 1% Triton X-100), and 300 μl phenol-chloroform, vortexed and centrifuged for 5 min at 5000 rpm. The supernatant was chloroform-extracted; 2.5X volume of ethanol absolute and a 0.1-volume of 3 M sodium acetate (pH 5.2) were added to the supernatant, and the tube was incubated at -20°C for 1 h followed by centrifugation for 10 min at 12,000 rpm. The precipitate was washed with cold 70% ethanol, dried in the air, and dissolved in 30 μL of distilled water. 

Extraction of DNA from clinical samples was performed as already described [ 16 ]. Briefly, a 50 μL (about 20 mg) of the specimen of the patient was transferred to a sterile 2 ml tub, containing a conical stainless steel bullet, cooled at -80 for at least 1 h, and shaken vigorously for 2 min. The bullet was washed with 100 μL of TE buffer (10 mM Tris, 1 mM EDTA) to reduce any sample loss, and the DNA purification was proceeded using a DNA purification kit (GeneAll, South Korea) and finally, 25 μL of elution buffer was added.

### Primer design

The reliable sequences of several molecular identification targets were downloaded from the National Center for Biotechnology Information (https://www.ncbi. nlm.nih. gov/pubmed/) (Table 2). They included the translation elongation factor 1-alpha (TEF-1α) [ 17 ], beta-tubulin [ 18 ], and internal transcribed spacer (ITS) region of ribosomal DNA (rRNA gene) [ 19 ] related to various species of dermatophytes and other common causative agents of superficial and cutaneous mycoses and some common environmental saprophytes.

**Table 2:** GenBank sequences of standard strains and clinical isolates used in this study for the analysis of translation elongation factor 1-alpha gene and internal transcribed spacer rDNA gene for primer designing

Translation elongation factor 1-alpha		
*Trichophyton rubrum*	*Trichophyton interdigitale*/*Trichophyton mentagrophytes*	*Epidermophyton floccosum*
MT448640.1	MT375512.1	MG356930.1
MH802505.1	MG356921.1	KM678060.1
MG251758.1	MG356901.1	MT448643.1
MF173062.1	MK460541.1	MG251796.1
KM678055.1	KM678130.1	MG356923.1
MT919256.1	MT375508.1	MG356928.1
MT872718.1	MG356914.1	MG251787.1
MG356893.1	MG356858.1	MG356927.1
MG251747.1	MG356908.1	MG356925.1
MT912005.1	MT375507.1	MG251779.1
**Internal transcribed spacer**		
*T. rubrum*	*T. interdigitale*/*T. mentagrophytes*	*E. floccosum*
CBS 392.58	MN691064.2	MT431956.1
MT188700.1	MK312848.1	MT040750.1
MT623559.1	MK447596.1	MN966495.1
MT131794.1	KP308373.1	MN808757.1
NR_131330.1	MN808775.1	MF434533.1
MT431172.1	MH790395.1	MF158309.1
MH791435.1	MK312828.1	NR_131275.1
MN460829.1	MZ044468.1	MT040763.1
MT191357.1	JN133969.1	MF158302.1
MT152325.1	MZ044458.1	AF168130.1

A careful primer selection for multiplex PCR application was done by Geneious software version 7 (http://www.geneious.com), assessing critical factors, such as compatibility of the primers. It should also be noted that the production of additional bands or spurious hybridizations of primer pairs to each other in amplification reactions was avoided. The oligonucleotide primers were synthesized by SinaClonCompany (SinaClon, Iran).

### Multiplex PCR

Multiplex PCR amplification was set up and performed on the DNA extracted from all fungal isolates and clinical samples under the following thermal conditions: 5 min at 95 °C followed by 35 cycles of 15 s at 95 °C, 30 s at 62 °C, and 20 s at 72 °C and a final extension step for 2 min at 72 °C. The reaction mixture contained 7.5 µl of 2X PCR premix (Ampliqon, Denmark), 10 pmol of each primer, 3 µl of DNA template, and enough water to reach a total volume of 15 µl. The PCR products were separated on 1.2% agarose gel, stained with ethidium bromide, and visualized under UV illumination. Appropriate positive and negative controls were included in each amplification reaction.

### Statistical analysis

Descriptive statistics were performed in SPSS software (version 11.0). Fisher exact test or chi-square test was used as required to compare categorical variables. A p-value of less than 0.05 was considered statistically significant. 

## Results

Species-specific primer pairs for *T. rubrum* (RubF–RubR) and *T. interdigitale*/*T. mentagrophytes* (IntF–IntR) were designed based on the sequence data for the TEF-1α gene, and the primers for *E. floccosum* (FloF–FloR) targeted the specific sequence of the ITS region. By using the designed specific primers in PCR reactions, sharp electrophoresis bands of approximately 360, 240, and 150 bp were seen for *T. rubrum*, *T. interdigitale*/*T. mentagrophytes*, and *E. floccosum*, respectively. These sizes are exactly the same as what was expected according to the in silico analysis of sequences used for primer designing.

Furthermore, other primers were developed at the sequence of TEF-1α for Microsporum canis (CanF-CanR) and Microsporum gypseum (GypF-GypR) as the most common zoophilic and geophilic dermatophytes, respectively, which are associated with tinea capitis and tinea corporis in human infection. Both analytical and clinical diagnostic performances of Can and Gyp primer pairs should be evaluated in the laboratory in the future. The selected primers and their predicted PCR product size are shown in Table 3.

**Table 3:** Species-specific primer pairs designed to amplify dermatophyte DNA

Target species	Gene region	PCR product	Primer name	Nucleotide sequences
*Trichophyton rubrum*	TEF-1α	358 bp	RubF	5'- ATCCCACTACAGGTGAAATTTTGG -3'
			RubR	5'- TGTTCCCTCATGTGGTTGTAC -3'
*Trichophyton mentagrophytes*/*Trichophyton interdigitale*	TEF-1α	235 bp	IntF	5'- CAGATTTGCTTTTTTCTGTCTTCAG -3'
			IntR	5'- CATCGTCTTGCTGTGCCGT -3'
*Epidermophyton floccosum*	ITS	147 bp	FloF	5'- TAGGCTGCAGTGTCGCTGCAGCG -3'
			FloR	5'- TACGAAATCTCCATAGGTGG -3'
*Microsporum canis*	TEF-1α	201 bp	CanF	5'- AGGCTGCTCTCTCTACCTTC -3'
			CanR	5'- TGCCTTGATGCTAATGAACC -3'
*Microsporum gypseum*	TEF-1α	172 bp	GypF	5'- ACATCAGGGATTTCAGCCAGAC -3'
			GypR	5'- TTGCTCTACATTCCCTTCTCCC -3'

PCR: polymerase chain reaction

**Figure 1:**
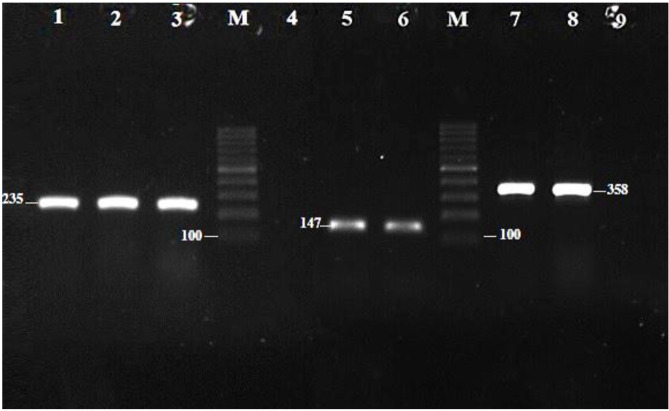
Multiplex polymerase chain reaction for examples of reference dermatophyte species. M: size markers (100 bp DNA ladder), line 1: Trichophyton mentagrophytes, line 2 and 3: Trichophyton interdigitale, line 4: negative control, line 5 and 6: Epidermophyton floccosum, line 7 and 8: Trichophyton rubrum, and line 9: negative control

The DNAs extracted from 101 fungal strains (24 *T. interdigitale*/*T. mentagrophyte*, 21 *E. floccosum*, 21 *M. canis*, 16 *T. rubrum*, 12 *T. tonsurans*, and 7 other fungi), which had already been identified by sequencing or PCR-RFLP, were used in a multiplex PCR assay. Table 1 summarizes the results of the multiplex PCR assays using specific primers designed in this study. All 24 T. interdigitale/T. mentagrophytes strains tested in this study yielded the expected product size. However, for 10 (83.3%) strains of T. tonsurans tested with the primer pairs selected for T. interdigitale/T. mentagrophyte (IntF–IntR), a band with the same size (235 bp) was observed. In total, 15 (93.7%) and 1 out of the 16 strains of T. rubrum tested by multiplex PCR were identified as T. rubrum strain and a mix of T. interdigitale/T. mentagrophyte and T. rubrum (with two sharp electrophoresis bands in 358 and 235 bp sizes) respectively. It should be mentioned that the PCR-RFLP results confirmed this mixture. In contrast to 21 strains that were identified as *E. floccosum* by sequencing or PCR-RFLP, multiplex PCR was able to amplify DNA in 20 (95.23%) strains and the 1 remaining strain was reported negative by this test. The multiplex PCRs were negative in 20 out of 21 strains that had been identified as M. canis, and 1 strain yielded an amplicon band related to RubF–RubR primers (358 bp). No PCR products were detected by the multiplex PCR performed for the seven non-dermatophyte fungal strains. The multiplex PCR results for different species of reference dermatophyte isolates are depicted in Figure 1.

**Figure 2:**
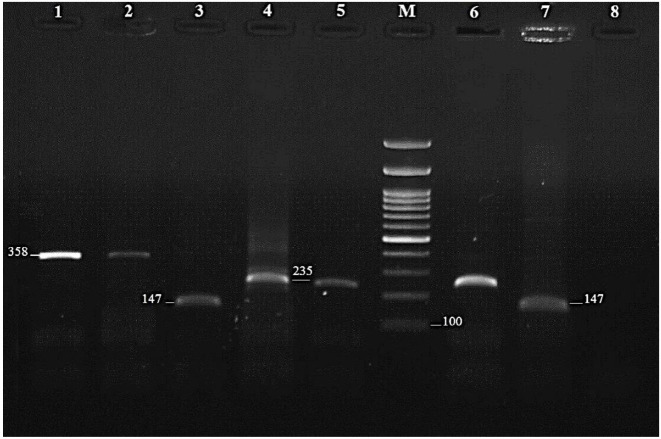
Multiplex polymerase chain reaction for examples of clinical samples suspected of cutaneous fungal infection. M: size markers (100 bp DNA ladder); lanes 1 and 2: Trichophyton rubrum; lanes 3 and 7: Epidermophyton floccosum; lanes 4, 5, and 6: Trichophyton interdigitale/Trichophyton mentagrophytes; and lane 8: negative control

In total, 155 DNAs were extracted directly from the clinical samples of patients suspected of cutaneous fungal infection. These DNAs were subjected to the multiplex PCR designed in this study to simultaneously detect the infection and identify the causative organisms. Multiplex PCR identified *T. rubrum* in 27 (17.4%) samples, *T. interdigitale*/*T. mentagrophytes*/*T. tonsurans* in 48 (30.9%) samples, *E. floccosum* in 7 (4.5%) samples, and 20 samples (13%) yielded multiple bands. For the 53 (34.2%) remaining samples, multiplex PCR was negative. Out of the twenty samples that had multiple bands, seven samples were a mixture of dermatophytes; one sample identified as a mix of *T. interdigitale*/*T. mentagrophytes* and *E. floccosum* (with two sharp electrophoresis band in 147 and 235 bp sizes) and six samples were mixed *T. interdigitale*/*T. mentagrophytes* and *T. rubrum* (with two sharp electrophoresis band in 358 and 235 bp sizes) and thirteen samples were unspecified. Examples of the multiplex PCR runs are presented in Figure 2.

Among 110 clinical samples for which both culture and multiplex PCR were performed, the proportion of samples with positive multiplex PCR (n=75, 68.1%) was higher than that of the samples with positive culture (n=61, 55.4%). It is noteworthy that this difference was statistically significant (P=0.001). Overall, it can be said that multiplex PCR was more sensitive than culture. In 52 (47.2%) samples, both multiplex PCR and culture were positive. In 32 (29%) samples, the results of multiplex PCR and culture were not concordant, accordingly, 17 and 6 out of 23 positive samples by multiplex PCR were detected negative and positive for non-dermatophyte mold or yeast by culture, respectively. Moreover, the remaining nine samples were negative by multiplex PCR and positive by culture. Finally, both multiplex PCR and culture were negative for dermatophytes in 26 (23.7%) samples (Table 4).

**Table 4:** Comparison of culture and multiplex PCR in terms of the detection/identification of 110 clinical samples suspected of cutaneous fungal infection

Clinical samples		Detected/identified by multiplex PCR					
		*Trichophyton mentagrophytes*/ *Trichophyton interdigitale*	*Trichophyton rubrum*	*Epidermophyton floccosum*	*T. interdigitale*/*T. mentagrophytes*/ *T. rubrum*	Negative	Total
Detected by culture	Positive for	Dermatophyte27	14	5	6	9	61
	Positive for non-dermatophyte fungi	1	2	0	3	6	12
	Negative	10	3	1	3	20	37
	Total	38	19	6	12	35	110

PCR: polymerase chain reaction

## Discussion

Efforts have been made to establish rapid and specific molecular-based techniques for species identification of the pathogenic dermatophytes, mainly based on primary isolation by culture. Sequence analysis of amplified ITS region is expensive and laborious; therefore, it is not easily employed for routine diagnostic purposes, particularly in low-income countries [ 20 , 21 ]. Likewise, real-time PCR is proved to be a sensitive and rapid but costly method for the identification of dermatophytes [ 11 , 22 ]. 

The present study aimed to design and evaluate a multiplex PCR technique that allowed simultaneous detection of three common major pathogenic dermatophytes within a working day: 2 h for preparation of DNA from culture isolates or clinical samples, 2 h for PCR amplification, and 1 h for electrophoresis. We were only interested in *T. rubrum*, *T. interdigitale*/*T. mentagrophytes*, and *E. floccosum* as they are the most common species isolated from dermatophytosis in humans [ 23 ]. 

In the present study, 1 (4.77%) isolate which had been identified as *E. floccosum* by sequencing or PCR-RFLP was negative in the multiplex PCR. The specific primers for *E. floccosum* were selected from ITS region which is known to be a genetic marker for the identification of dermatophytes species [ 24 ]. This might be since some DNAs were old samples left for a few years. No ITS sequence suitable for designing specific primers for *T. rubrum*, and *T. interdigitale*/*T. mentagrophytes* species could be found. Hence, the TEF-1α region (that has been introduced as a suitable gene for identification of some complexes, such as *Arthroderma vanbreuseghemii*, *T. rubrum*, *Arthroderma benhamiae*, and *Arthroderma otae* [ 17 ]) was selected for primer selection. 

Nevertheless, designed multiplex PCR containing the primer pair Int from TEF-1α region cross-reacted with *T. tonsurans*. This is not unexpected since there is a high degree of genetic similarity in the TEF-1α region between the *T. mentagrophytes* complex and *T. tonsurans* [ 17 ]. However, the TEF-1α length variation between *T. interdigitale* and *T. tonsurans* strains (10–25 bp) was found to be significantly higher than that of other loci like the ACT, TOP-II, ITS, and BT2 [ 25 - 27 ]. Regardless of the results of *T. tonsurans* isolates, the specificity of the technique was good as neither the other dermatophytes species nor the non-dermatophyte molds and yeasts yielded positive results in multiplex PCR. 

The collected data demonstrated that multiplex PCR test is as sensitive as traditional diagnostic methods if culture-positive samples are considered true positives (47.3% positive by both tests). As shown in Table 4, multiplex PCR was negative for nine samples that were culture-positive for dermatophyte species. A likely reason for these negative results is that the causal agents of dermatophytosis in these samples were species other than those considered in this multiplex PCR. Another reason might be that the positive material was not contained in the subsample set aside for molecular testing. It should be noted that such problems with sample division have long been a known factor in dermatologic mycology testing [ 28 ]. In total, 23 culture-negative samples were positive in multiplex PCR. Negative culture results of patients with dermatophytosis could be due to prior medical treatments; hence, these cases should always be investigated further.

Although culture did not identify any mixed dermatophyte infection among the samples in this study, multiplex PCR co-detected *T. rubrum* and *T. interdigitale* in 12 samples. Sampling variation is a more likely explanation for this finding as the culture needed multiple pieces of sample to yield the growth of both dermatophytes. Another explanation is that if multiple dermatophyte species are present in a sample, in culture, the predominant dermatophyte is likely to outperform the less abundant one.

This multiplex assay detects three out of several causal agents of dermatophytosis; therefore, it cannot be used as a comprehensive diagnosis/identification test. However, it is valuable for two phenotypically similar species i.e., *T. rubrum* and *T. interdigital*, as the most common dermatophytes all around the world and an easy-to-use tool in outbreak investigations. However, this defect can be eliminated by the addition of primers targeting the pan-dermatophyte-specific sequence or the addition of more specific primers for the detection of more species.

## Conclusion

In this study, a multiplex PCR was presented using specific primers as a rapid and accurate method for the identification of the three most common pathogenic dermatophytes, not only from cultured colonies but also directly from the clinical samples. Despite its limitations, this multiplex PCR looks robust and can be easily run in a routine laboratory with obvious advantages, such as markedly reduced diagnosis time and higher sensitivity.

Authors’ contribution

S.F. and Sh.A. performed the experimental works. A.RM., S.A., M.D. and, M.M. contributed to data collection. M.B. Participated in the design of the study. M.M. wrote the draft version of the article. H.M. supervised all parts of the study and writing the paper.

Financial disclosure

There are no financial conflicts of interest to disclose.

## References

[1] Ahmad Khan MS, Ahmad I ( Cambridge, MA: Academic). Chapter 1-herbal medicine: current trends and future prospects. New look to phytomedicine.

[2] Ginter‐Hanselmayer G, Weger W, Ilkit M, Smolle J ( 2007). Epidemiology of tinea capitis in Europe: current state and changing patterns. Mycoses.

[3] Naseri A, Fata A, Najafzadeh MJ, Shokri H ( 2013). Surveillance of dermatophytosis in northeast of Iran (Mashhad) and review of published studies. Mycopathologia.

[4] AL-Khikani FH ( 2020). Dermatophytosis a worldwide contiguous fungal infection: growing challenge and few solutions. Biomed Biotechnol Res J.

[5] Leung AK, Lam JM, Leong KF, Hon KL ( 2020). Tinea corporis: an updated review. Drugs Context.

[6] Dragos V, Lunder M ( 1997). Lack of efficacy of 6‐week treatment with oral terbinafine for tinea capitis due to Microsporum canis in children. Pediatr Dermatol.

[7] Fleece D, Gaughan JP, Aronoff SC ( 2004). Griseofulvin versus terbinafine in the treatment of tinea capitis: a meta-analysis of randomized, clinical trials. Pediatrics.

[8] Lee MK, Kim HR, Lee YJ ( 2006). Identification of Candida species by multiplex polymerase chain reaction. Korean J Clin Microbiol.

[9] Kim JY, Choe YB, Ahn KJ, Lee YW ( 2011). Identification of dermatophytes using multiplex polymerase chain reaction. Ann Dermatol.

[10] Dhib I, Fathallah A, Yaacoub A, Hadj Slama F, Said M, Zemni R ( 2014). Multiplex PCR assay for the detection of common dermatophyte nail infections. Mycoses.

[11] Arabatzis M, Bruijnesteijn van Coppenraet L, Kuijper E, De Hoog G, Lavrijsen A, Templeton K, et al ( 2007). Diagnosis of common dermatophyte infections by a novel multiplex real‐time polymerase chain reaction detection/identification scheme. Br J Dermatol.

[12] Ross IL, Weldhagen GF, Kidd SE ( 2020). Detection and identification of dermatophyte fungi in clinical samples using a commercial multiplex tandem PCR assay. Pathology.

[13] Rezaei-Matehkolaei A, Makimura K, de Hoog S, Shidfar MR, Zaini F, Eshraghian M, et al ( 2013). Molecular epidemiology of dermatophytosis in Tehran, Iran, a clinical and microbial survey. Med Mycol.

[14] Rezaei-Matehkolaei A, Makimura K, Shidfar M, Zaini F, Eshraghian M, Jalalizand N, et al ( 2012). Use of single-enzyme PCR-restriction digestion barcode targeting the internal transcribed spacers (ITS rDNA) to identify dermatophyte species. Iran J Public Health.

[15] Makimura K, Mochizuki T, Hasegawa A, Uchida K, Saito H, Yamaguchi H ( 1998). Phylogenetic classification of Trichophyton mentagrophytes complex strains based on DNA sequences of nuclear ribosomal internal transcribed spacer 1 regions. J Clin Microbiol.

[16] Motamedi M, Mirhendi H, Zomorodian K, Khodadadi H, Kharazi M, Ghasemi Z, et al ( 2017). Clinical evaluation of β‐tubulin real‐time PCR for rapid diagnosis of dermatophytosis, a comparison with mycological methods. Mycoses.

[17] Mirhendi H, Makimura K, de Hoog GS, Rezaei-Matehkolaei A, Najafzadeh MJ, Umeda Y, et al ( 2015). Translation elongation factor 1-α gene as a potential taxonomic and identification marker in dermatophytes. Med Mycol.

[18] Mirhendi H, Motamedi M, Makimura K, Satoh K ( 2016). Development a diagnostic pan‐dermatophyte TaqMan probe real‐time PCR assay based on beta tubulin gene. Mycoses.

[19] Jackson CJ, Barton RC, Evans EG ( 1999). Species identification and strain differentiation of dermatophyte fungi by analysis of ribosomal-DNA intergenic spacer regions. J Clin Microbiol.

[20] Kong F, Tong Z, Chen X, Sorrell T, Wang B, Wu Q, et al ( 2008). Rapid identification and differentiation of Trichophyton species, based on sequence polymorphisms of the ribosomal internal transcribed spacer regions, by rolling-circle amplification. J Clin Microbiol.

[21] Li HC, Bouchara JP, Hsu MM, Barton R, Su S, Chang TC ( 2008). Identification of dermatophytes by sequence analysis of the rRNA gene internal transcribed spacer regions. J Med Microbiol.

[22] Sherman S, Goshen M, Treigerman O, Ben‐Zion K, Carp MJ, Maisler N, et al ( 2018). Evaluation of multiplex real‐time PCR for identifying dermatophytes in clinical samples-A multicentre study. Mycoses.

[23] Ebrahimi M, Zarrinfar H, Naseri A, Najafzadeh MJ, Fata A, Parian M, et al ( 2019). Epidemiology of dermatophytosis in northeastern Iran; A subtropical region. Curr Med Mycol.

[24] Suh MK, Kim BC, Kim JC ( 2000). Phylogeny and taxonomy of the dermatophytes using sequence analysis of the ribosomal internal transcribed spacer 1 region. Korean J Dermatol.

[25] Graser Y, Kuijpers A, Presber W, Hoog GD ( Med Mycol J 1999). Molecular taxonomy of Trichophyton mentagrophytes and T. tonsurans.

[26] Ahmadi B, Mirhendi H, Makimura K, de Hoog GS, Shidfar MR, Nouripour-Sisakht S, et al ( 2016). Phylogenetic analysis of dermatophyte species using DNA sequence polymorphism in calmodulin gene. Med Mycol.

[27] Rezaei-Matehkolaei A, Mirhendi H, Makimura K, de Hoog GS, Satoh K, Najafzadeh MJ, et al ( 2014). Nucleotide sequence analysis of beta tubulin gene in a wide range of dermatophytes. Med Mycol J.

[28] Wiegand C, Bauer A, Brasch J, Nenoff P, Schaller M, Mayser P, et al ( 2016). Are the classic diagnostic methods in mycology still state of the art?. J Dtsch Dermatol Ges.

